# Is Violent Radicalisation Associated with Poverty, Migration, Poor Self-Reported Health and Common Mental Disorders?

**DOI:** 10.1371/journal.pone.0090718

**Published:** 2014-03-05

**Authors:** Kamaldeep Bhui, Nasir Warfa, Edgar Jones

**Affiliations:** 1 Wolfson Institute of Preventive Medicine, Queen Mary University of London, London, United Kingdom; 2 King’s Centre for Military Health Research, King’s College London, London, United Kingdom; Xi’an Jiaotong University School of Medicine, China

## Abstract

**Background:**

Doctors, lawyers and criminal justice agencies need methods to assess vulnerability to violent radicalization. In synergy, public health interventions aim to prevent the emergence of risk behaviours as well as prevent and treat new illness events. This paper describes a new method of assessing vulnerability to violent radicalization, and then investigates the role of previously reported causes, including poor self-reported health, anxiety and depression, adverse life events, poverty, and migration and socio-political factors. The aim is to identify foci for preventive intervention.

**Methods:**

A cross-sectional survey of a representative population sample of men and women aged 18–45, of Muslim heritage and recruited by quota sampling by age, gender, working status, in two English cities. The main outcomes include self-reported health, symptoms of anxiety and depression (common mental disorders), and vulnerability to violent radicalization assessed by sympathies for violent protest and terrorist acts.

**Results:**

2.4% of people showed some sympathy for violent protest and terrorist acts. Sympathy was more likely to be articulated by the under 20s, those in full time education rather than employment, those born in the UK, those speaking English at home, and high earners (>£75,000 a year). People with poor self-reported health were less likely to show sympathies for violent protest and terrorism. Anxiety and depressive symptoms, adverse life events and socio-political attitudes showed no associations.

**Conclusions:**

Sympathies for violent protest and terrorism were uncommon among men and women, aged 18–45, of Muslim heritage living in two English cities. Youth, wealth, and being in education rather than employment were risk factors.

## Introduction

Studies of 9/11 show that as well as death and wounding caused by acts of terrorism, health risks include chronic health problems among emergency service workers and among exposed populations. [1], [2] Terrorism is designed to promote fear and anxiety, whilst direct experience of terrorist incidents can cause post-traumatic disorders in adults and young people. [3], [4] Furthermore, over-reaction or extreme counter-terrorism responses can become restrictive and erode individual freedoms and the bonds of a democratic society. [5], [6] The indirect consequences include social divisions between diverse religious and cultural groups, and the undermining of social cohesion with significant implications for health. [7] Once terrorists are captured, there is often a debate about what motivated their behaviour, whether they came from disadvantaged backgrounds, have predisposing psychiatric disorders, and whether their acts were purely political. These issues are considered during criminal justice and forensic psychiatry assessments. For example, Victoroff has argued that depression is a component in the process of radicalisation [8], an important finding for both doctors and lawyers.

A great deal of effort and significant financial resources are committed to counter-terrorism. There is less attention given to researching preventive interventions. Health practitioners and local government officials have targeted violence in the community, including domestic violence, gun crime, as well as suicide and bio-terrorism. [9]–[11] A similar approach has not been applied to radicalisation, perhaps because this requires a better understanding of who is at risk of developing these sympathies. Silber and Bhat argue that radicalisation is a staged process that starts with pre-radicalisation and moves through stages of self-identification, indoctrination, and finally Jihadization. [12] We hypothesize that a preventive intervention needs to interrupt the ‘pre-radicalisation’ phase, a period when individuals begin to develop sympathies for extremist ideas or terrorist movements without becoming directly involved. This is an accord with the preventive approach found in public health in which a common antecedent of vulnerability to future illness or risk behaviours is targeted for intervention. A better understanding is required of personal and situational characteristics that are markers of this early phase of risk [13], [14].

One of the challenges is the absence of a measure of the early stages of radicalisation. Most previous measures were developed and used in the Middle-East or in Muslim majority countries and are designed to assess the extent of established commitment to extreme terrorist action, rather than the early phases of radicalisation that include the emergence of sympathies for violent protest and terrorist causes. [15]–[21] Other studies developed and tested tools to assess terrorists in contact with forensic services. [18], [20], [21] The Belief Diversity Scale, for example, asked about extreme ‘Middle Eastern ideologies’, including 33 items about attitudes to Israel, women, politics, religiosity and the use of religion to recruit terrorists, the West, and fighting for a cause. [22]–[24] Kennedy et al (2008) developed a 47 item measure of behaviours, attitudes and identity issues indicative of terrorist intentions; this study was based on among 33 ‘insiders’ who were considered to be ‘critics’ of Islam, ‘defenders’, or ‘mainstream’ Muslims. [18] Interestingly, the three groups expressed different levels of concern and worry about the same behaviours and attitudes. ‘Defenders’ were more likely to understate concern, and Muslims who were ‘critics’ of the fundamental teachings of Islam were more worried. Schbley (2003) used convergence of psychometric measures from 356 suicide-bombers, taped self-immolations of 15 terrorists and 918 ‘zealots’ to triangulate the data in order to isolate relevant risk factors in a mixed-method design. [19] This produced a 32-trait profile of a ‘religious terrorist’, an ethno-religious specific description. The evidence identified the following risk markers: predisposition to violence in the name of Allah, susceptibility to joining a cult, susceptibility to dogma-induced psychotic depression and affinity for martyrdom. This study was novel in that it investigated psychiatric symptoms and implicated psychotic depression and personality factors. A new risk assessment instrument developed in Canada focused on convicted terrorists. It assessed characteristics shared by terrorists from diverse backgrounds: [20], [21] ideologies, affiliations and moral emotions, grievances about perceived victimization and injustice, contextual factors such as contact with other extremists or isolation, previous exposure to violence, and commitment to acts of terrorism because of anger or ideology about the rewards of participating in terrorism. In summary, these studies included survey questions and forensic assessment methods. Most were conducted in Muslim majority countries rather than in Western democracies. None studied health status or explored the early phase of radicalisation in populations.

Because the perpetrators of many recent, high-profile terrorist attacks were citizens who worked and were educated in the countries that they attacked, a core issue for prevention is how to identify people who have no history of criminal behaviour but are motivated to commit acts of terrorism. [25] For example, on April 2013, two men bombed the Boston marathon resulting in three deaths and injuries to 264 spectators and runners. They both appeared to have been integrated within US society. [26] The murder of Fusilier Lee Rigby on 22 May 2013 was widely reported in the media and by the UK government as a terrorist incident. It involved two young men, both born in the UK to Christian families and educated at the University of Greenwich. Having been converted to a radical form of Islam, they planned the killing of a British serviceman to highlight their opposition to UK foreign policy, which they perceived as a threat to Muslim countries [27].

Radicalisation is the construct proposed to explain this phenomenon. It is defined as a social and psychological process by which ordinary citizens become so aggrieved that they are willing to sacrifice their lives and the lives of innocent civilians to make a political protest. [25] Much that is written about radicalisation is based on the biographies of convicted terrorists and those on de-radicalisation programmes [25], [28]–[30]. These histories are re-constructed and characteristics identified during interrogation are uncritically assumed to be of relevance to the early phase of radicalisation. Criminality, poor health, depressive symptoms, risky behaviour in young men, social inequalities, personality variables, international foreign policy and social networks have all been proposed to be influential drivers for grievances that lead to radicalisation. [7] In reality, little empirical research has been conducted into the early stages of radicalisation. It is still unclear what factors make potential recruits open to persuasion to join a political movement that incites violent protest and terrorism. This ‘open-to-persuasion’ phase is marked by growing sympathies with terrorist organizations and political causes that endorse the use of violence [12].

In this paper we take an interdisciplinary perspective, testing health, social, and political influences on vulnerability to radicalisation. We define radicalisation in accord with the British Terrorism Act of 2000 as the process by which a person comes to support terrorism and forms of extremism leading to terrorism. [25] Violent extremism is described as endorsement of violence to achieve extreme ends [25].

- This paper describes the development of a new measure of radicalisation that is based on sympathies for violent protest and terrorism among men and women of Bangladeshi and Pakistani origin and with a Muslim heritage.
- We assess the population prevalence of sympathies towards violent protest and terrorism, and investigate the relationships with poor self-rated health, anxiety and depression, and other hypothesised determinants of radicalisation, for example, poor political engagement, low social capital, adverse life events, poverty, and migrant status.

## Materials and Methods

Ethical approval was from Queen Mary, University of London Research Ethics Committee. The study was also subject to an independent monitoring committee chaired by a professor of ethics in the law department at Queen Mary University of London. No adverse incidents or issues were reported during data collection. The work was undertaken with the support and advice of a public involvement panel of local and national community organisations with expertise and interest in the subject, including representative of mosques, students, and health agencies.

### Measuring Radicalisation

To develop a new measure of radicalisation, we consulted Muslim and non-Muslim researchers and members of local community panels (consisting of local charities and mental health and educational organizations and religious institutions) about how to measure radicalisation. This phase of enquiry orientated our study within local and national sentiments and sensitivities. We then held focus groups including people who spoke English and were of Muslim heritage. They were selected as the study focused on people who appear to be integrated in UK society rather than recent migrants, in order to meet the objectives of the main study (see Table 1). Snowballing was used to assemble the focus group and aimed to draw people of Muslim heritage, and with a background in mental health or social science or public health, in order to formulate psychological and social-science constructs, together with ‘insider’ cultural insights and perspectives on radicalisation. This was necessarily a purposive rather than a representative sample, but was suited to the purpose of the focus group; that is we aimed to identify suitable items for a new measure of radicalisation, and to test the acceptability and face and content validity of the survey questions. We did not include criminal justice representatives in the focus groups because the current knowledge and literature has already been informed by such influences. In addition, we were advised that their involvement at this stage might inadvertently constrain the freedom with which focus group participants would offer their views and would undermine our intention of eliciting community perspectives. However, in our wider consultations we discussed the study design with experts on terrorism, security and counter-terrorism policy. This also assured compliance with legal requirements.

**Table 1:** Demographics of Focus Group 1.

Gender	Ethnicity	Nationality	Occupation
Female	Bangladeshi	British	Psychologist
Male	Pakistani	British	Psychiatrist
Male	Iraqi	British	Psychiatrist
Female	Turkish	Turkish	Student
Female	Turkish	Turkish	Student
Female	Turkish	Turkish	Student
Female	Turkish	Turkish	Student
Male	Black African	British	IT Professional
Male	Black African	British	Mental Health Professional
Female	Iranian	Iranian	Student

Age Range 22–56.

There were two focus group facilitators: one of Somali Muslim background (NW) and one of South Asian and non-Muslim background. No financial incentives were offered to any participants. A brief demographic questionnaire was completed and all participants provided consent. Participants suggested specific items to measure radicalization, and commented on the suitability of a draft survey questionnaire, whilst dismissing some items if they were too sensitive, lacked clarity, or if they did not reflect an authentic Islamic perspective and might be misunderstood. The focus group settled on seventeen questions as a measure of radicalisation. These were piloted and tested in 8 individual pilot interviews, in which subjects were debriefed and asked how they felt answering the questions. Modifications to the items and removal of one item as a consequence resulted in a 16-item inventory of radicalisation (called the SyfoR), based on asking subjects about sympathies for or condemnation of 16 different actions The items reflected a range of actions that fell under the heading of violent protest, violent radicalisation and terrorism; these included sympathies for use of suicide bombs to fight injustice or commit terrorist acts as a form of political protest.

### Survey Sampling

The study included 608 people of Pakistani or Bangladeshi origin men and women, aged 18–45, of Muslim heritage and living in East London and Bradford. These cities were chosen because they are home to a significant Muslim population of Bangladeshi and Pakistani origin in areas of contrasting deprivation and cultural integration with wider society. [31] Individuals were recruited by proportional quota sampling. This is a standard method that entails setting quotas for participants on a range of demographic factors and ensures that the sample interviewed is representative of the population of interest. Individuals living within specific households within a sampling unit were identified by door knocking and offered a computer assisted interview if they gave informed consent. Flash cards were used to simplify the process of answering questions with choices. Quota sampling offers an alternative to probability sampling and is often used in market research and national surveys [32]–[34], and becomes necessary if there is no listing of all those eligible to be included. It is more efficient as recruitment and sampling can be focused in areas in which the desired population are resident but does require good census data on the characteristics by which the quota are set. This method is preferred if the costs of probability sampling would be prohibitive and where feasibility issues become prohibitive. Given the sensitivity of the survey and the specific sampling criteria, we did not wish to expose large numbers of people who would not meet our inclusion criteria to the preliminary recruitment phase. Using UK Census 2001 data, quotas were set for each sampling unit to reflect the key demographic variables of those living there. Target quotas were set for age (18–30 years and 31–45 years) gender, work status (working full-time, not working full-time) and ethnicity (Pakistani and Bangladeshi). These quotas were based on the expected number of Muslim households in each output area (London and Bradford), which was estimated using the 2001 Census and mid-year 2010 estimates. Local areas were then classified into a high, medium, or low concentration areas. Muslim households were over-sampled from high and medium concentration areas to ensure recruitment was efficient. Interviewers were permitted to select any eligible resident from within the sampling unit to meet their quotas, but a minimum of three houses were left between each attempt to interview and only one interview per household was permitted. The actual number of interviews per sampling unit closely matched, and mostly exceeded the expected numbers that were set ‘a priori’; only for Pakistanis recruited from East London was there a lower number of actual compared with expected numbers (5 rather than 10). Overall design effect due to sampling within different areas of Muslim household concentration and non-response weights (expected population proportion/actual proportion) were provided by IPSOS-MORI in order to generate population level estimates of prevalence. The weights were capped at the 97^th^ percentile to reduce the impact of a few extreme values, and the weights were rescaled so that the weighted total equalled the unweighted total number of interviews.

### Measuring Sympathies for Violent Radicalisation & Terrorism

During the survey, subjects were asked to rate their support for or condemnation of the 16 items of the instrument that we have called the SyfoR. The responses were in the form of a 7-item Likert scale with scores of −3 to +3. For all except two of these, a higher score indicated greater support for violent protest, radicalisation and terrorism. These two items, which asked about sympathies for or condemnation of the British government’s decision to send British troops to Afghanistan and Iraq, were reverse-scored as condemnation reflected a more radicalized perspective.

### Demographic, Social and Health Characteristics

Questions to assess psychosocial and health risk were also reviewed by the focus group and by the pilot interviews and questions modified to make them acceptable and easily understood. As a measure of social support and strength of social networks, we asked about the number of contacts by telephone, email, or visit in the preceding two weeks by friends or relatives. We asked about the proportion of the subject’s friends that shared his or her own ethnic background (*all* or *a lot*, *about half*, *none* or *very few*). Social capital is known to be related to violence, [35], [36] suicide [37] and mental health. [38] Given the constraints in the length of the survey, we selected questions a number of questions from the Office for National Statistics Social Capital Question Bank [39] in order to tap the most important elements of social capital of relevance to radicalisation. We attempted to ensure our methods are replicable and comparable with other studies using the same questions. Therefore, to assess social capital, we asked three questions about satisfaction with living in the area (very satisfied, fairly satisfied, neither, fairly dissatisfied, very dissatisfied), trust in neighbours (may people, some people, a few, none), and feelings of safety (very safe, fairly safe, fairly unsafe, very unsafe). These were scored (higher score meaning more social capital) and summed. The results are presented by total score and by each item.

The questions to assess political engagement were drawn from the Department of Communities and Local Government Citizenship Survey. These asked whether individuals had voted in the last local council election, discussed politics or political news with someone else, signed a petition, donated money to a charity or campaigning organisation, paid a membership fee to a charity or campaigning organisation, done voluntary work, boycotted certain products (for political, ethical or environmental reasons), boycotted certain products for religious reasons, expressed my political opinions online, been to any political meeting, donated money or paid membership fees to a political party and taken part in a demonstration, picket or march. The total number of activities formed a measure of political engagement (scores 0–12).

Discrimination was assessed by questions from the EMPIRIC study asking about physical assault, damage to property, insults, unfair treatment at work, job refusal due to race, religion or culture (score 0–5 for each item endorsed; total score 0–25). [40] Life events were assessed by the 12 items from the threatening life events inventory (score 0–12). [41] We also asked about emergency department attendance or recent injuries, as indicators of risky behaviour.

To measure general functioning related to health, four questions used in the ‘Health & wellbeing survey of serving and ex-serving members of the UK Armed Forces’ and adapted from the SF12 were used. [42], [43] These self-report measures assessed global general health (fair, poor, good, very good, excellent), reduced social activities because of physical or emotional problems (a great deal, a fair amount, a little, not at all), cut down on work and other activities because of physical or emotional health (a great deal, a fair amount, a little, not at all), and not being able to undertake any vigorous activity because of health problems (no, a little, a lot). These were each recoded to binary variables (poor/fair health vs. rest; not at all/a little vs. rest; not at all/a little vs. rest; no vs. rest respectively). These binary scores were summed giving a total score (0–4).

Anxiety was measured by the Generalised Anxiety Disorder Assessment (GAD-7) [44] and depression by the Patient Health Questionnaire (PHQ-9) as total scores. [45] We asked about age (18–20, 21–25, 26–30, 31–35, 36–40, 41–45), gender, marital state (single or not), employment (employed, full-time education, unemployed, at home for another reason such as retirement, disability or looking after the house), personal annual income, country-of-birth (UK or not). One question on religion asked about the frequency of attending a place of worship (never, monthly or less, weekly or more).

### Data Collection

The data collection was undertaken by IPSOS-MORI, an experienced social research organisation, which has previously run community surveys of sensitive topics. Early pilot work indicated matching by language was not necessary as the population of interest were English speakers and the interviewers had much experience of cross-cultural and cross-religious surveys, having been involved in previous studies. It also has a workforce of trained interviewers resident in the communities where the fieldwork took place. Questions were asked in a computer-assisted format with prompts and cues so that sensitive questions could be answered anonymously. Individual consents were secured at the beginning of the interview. Two sets of four interviews were undertaken to pilot the questions. After each set, interviewees were de-briefed and asked if they had understood the questions, and if they had any recommendations to improve the flow of question and reduce the burden to respondents. This process refined the questionnaire so that the interviews could be completed within 30 minutes, and served to improve the content and face validity of the survey, as respondents were asked if concepts were not understood or did not make sense to them. These pilot interviews were not included in the main study sample.

### Data Analysis

The items of the sympathies for radicalisation scale were subjected to pairwise correlations (Spearman’s alpha) and inter-item reliability tests (alpha), The raw scores are described without weights, for each of the Likert options (−3 to +3) as well as by three categories: any condemnation (−3 to −1), any sympathies (+1 to +3), and neither (score of 0). If subjects did not know or were uncertain they were coded as scoring 0. The items were then subjected to a principal components factor analysis, with an orthogonal rotation, to assess the factor structure of the new measure, and to see if the items relating to non-violent and violent protest, and terrorist actions were located within the same or distinct factors.

The total score was calculated on the basis of the items contributing to the factors that showed relevance following the factor analysis. The score was then recoded into a binary variable (score of −1 or less, versus scores of 0 or more) in logistic regression models. Score of 0 or more were considered to reflect those vulnerable to radicalisation. Univariate and multivariate models included the potential explanatory factors. The multivariate logistic regression model was built including age and gender, and the variables that were significantly associated with being *vulnerable to radicalisation* in the univariate analyses (at p = 0.05 level). Only weighted models are shown, as the findings are generalisable to the Muslim population from which the participants were drawn. Odds ratios, 95% confidence intervals, p values and R^2^ values were presented.

## Results

Pair-wise Spearman correlation coefficients were calculated (see Table 2) showing that sympathies for non-violent protest were negatively correlated with all other items that asked about sympathies for violent protest. The items asking about British troops going to fight in Iraq and Afghanistan – reflecting attitudes to international conflict-were also negatively correlated with items on violent protest. The main items not related to international conflicts and non-violent protest otherwise showed good face validity.

**Table 2:** Spearman correlation coefficients between items of SyfoR (unweighted raw data).

		nvp	vpf	vpt	vfp	vfg	mc	pv	thrta	Org	ta	bo	sbo	ttA	ttl	ftA	ftl
non-violent protest	nvp	*1.000*															
violence to protect family	vpf	***0.282***	*1.000*														
violence to protect tribe	vpt	*0.003*	***0.389***	*1.000*													
violence to fight police injustice	vfp	***−0.130***	***0.383***	***0.667***	*1.000*												
violence to fight government injustice	vfg	***−0.183***	***0.363***	***0.639***	***0.859***	*1.000*											
minor crime	mc	***−0.093***	***0.221***	***0.421***	***0.487***	***0.516***	*1.000*										
political violence	pv	***−0.224***	***0.165***	***0.434***	***0.549***	*0.579*	***0.676***	*1.000*									
threaten terrorist actions	thrta	***−0.223***	***0.124***	***0.423***	***0.496***	***0.533***	***0.575***	***0.763***	*1.000*								
organise radical groups	org	***−0.228***	***0.130***	***0.408***	***0.521***	***0.532***	***0.552***	***0.719***	***0.803***	*1.000*							
involved in terrorist actions	ta	***−0.247***	***0.116***	***0.380***	***0.470***	***0.498***	***0.486***	***0.673***	***0.815***	***0.816***	*1.000*						
use of bombs	bo	***−0.253***	***0.116***	***0.413***	***0.537***	***0.574***	***0.467***	***0.612***	***0.697***	***0.662***	***0.689***	*1.000*					
use of suicide bombs	sbo	***−0.251***	***0.119***	***0.393***	***0.537***	***0.556***	***0.470***	***0.603***	***0.699***	***0.662***	***0.671***	***0.896***	*1.000*				
British troops to Afghanistan	ttA	***−0.035***	***−0.109***	***−0.310***	***−0.291***	***−0.326***	***−0.214***	***−0.289***	***−0.349***	***−0.338***	***−0.334***	***−0.386***	***−0.439***	*1.000*			
British troops to Iraq	ttI	***−0.010***	***−0.113***	***−0.323***	***−0.313***	***−0.345***	***−0.226***	***−0.294***	***−0.363***	***−0.331***	***−0.331***	***−0.392***	***−0.450***	***0.894***	*1.000*		
People going to fight British troops inAfghanistan	ftA	*0.068*	***0.220***	***0.352***	***0.397***	***0.421***	***0.283***	***0.365***	***0.433***	***0.420***	***0.442***	***0.404***	***0.395***	***0.337***	***0.357***	*1.000*	
People going to fight British troops inIraq	ftI	*0.039*	***0.226***	***0.349***	***0.407***	***0.428***	***0.262***	***0.364***	***0.421***	***0.404***	***0.435***	***0.426***	***0.426***	***0.359***	***0.356***	***0.914***	*1.000*

Bold font = significant to a p = or <0.01 level.

A principal components factor analysis with an orthogonal rotation to improve fit showed four main factors (Table 3) which we have named, radicalisation, defensive violence, going to another country to fight British troops, and sending British troops to another country. Thus, taken with the Spearman’s correlations, the items relating to radicalisation and defensive violence were well correlated showing good face and content validity; they belong to factors 1 and 2. Factors 3 and 4 relate to British people going to fight in other parts of the world and with UK forces or British soldiers going to fight in other parts of the world. These were intended to reflect attitudes to international policy, but seemed to be separate factors and so were not used; furthermore, the negative correlations of items in factor 4 seemed to suggest that they way subjects had addressed these questions was not consistent with our original framework of a positive score reflecting sympathies for forms of violent protest.

**Table 3:** Principal components analysis of the 16 items developed through focus groups to measure radicalisation *(orthogonal rotated solution presented)* (obs = 575. 58 parameters).

	Rotated factor loadings			
Actions as part of political protests	F1Radicalisation	F2DefensiveViolence	F3Brit. CitizensFighting UK	F4ForeignPolicy
*Take part in non-violent protest*	***−***0.519	0.257	0.264	***−***0.141
*Commit minor crime*	***0.5233***	***0.452***	***−***0.023	***−***0.055
*Use violence*	**0.728**	0.34	0.079	***−***0.075
*Threaten to commit terrorist acts*	**0.812**	0.161	0.235	***−***0.159
*Organise radical terrorist groups without personally taking part*	**0.783**	0.191	0.222	***−***0.116
*Commit terrorist acts*	**0.805**	0.128	0.266	***−***0.138
*Use of bombs to fight injustice*	**0.785**	0.135	0.204	***−***0.185
*Use of suicide bombs to fight injustice*	**0.758**	0.114	0.185	***−***0.285
*Violence to protect family*	***−***0.144	**0.72**	0.189	***−***0.006
*Violence by organised groups to protect own race/religious* *group or tribe*	0.201	**0.719**	0.133	***−***0.182
*Violence to fight police injustice*	0.391	**0.743**	0.165	***−***0.091
*Violence to fight government injustice*	0.426	**0.701**	0.171	***−***0.129
*People in Britain who went to fight against UK in Afghanistan*	0.208	0.145	**0.911**	***−***0.139
*People in Britain who went to fight against UK in Iraq*	0.226	0.125	**0.903**	***−***0.127
*Government’s decision to send UK soldiers to Afghanistan* *	***−***0.141	***−***0.071	***−***0.114	**0.947**
*Government’s decision to send UK soldiers Iraq* *	***−***0.151	***−***0.083	***−***0.123	**0.943**
Variance	4.719	2.623	2.11	2.08
Proportion	0.295	0.164	0.132	0.13
Cumulative	0.295	0.459	0.591	0.721

*indicates reverse scored so sympathies are always consistently towards radicalisation.

We used the 11 items related to factors 1 and 2 as a measure of sympathies for radicalisation (or SyfoR). The inter-item reliability coefficient was 0.89, with an inter-item covariance of 0.85. The total score produced by summing the scores of these 11 items was used as a conceptually coherent radicalisation scale with good face and content validity and internal reliability. The scores ranged from ***−***33 to 21. Thirteen individuals had a positive score (2.4%), 39 scored zero (6.41%), and the remainder had a negative score. Of those with positive scores, one individual scored 21, one scored 10, one scored 7, and then two people each scored 1,2,3,4,5 or 6. Given that the actions described in the items are violent, we grouped those scoring 0, 1 or more as individuals who might be considered vulnerable to radicalizing influences; that is, they did not show outright condemnation of such acts and showed sympathy or uncertainty. Using this binary outcome, logistic regression analyses (weighted for clustering and non-response) were completed for demographic, cultural, social, and health variables (see Table 4).

**Table 4:** Logistic Regression Models showing univariate relationship with radicalisation outcome.

Characteristics	Level	N	Univariate			
			OR	95% CI	p	R^2^
**Age**	18–20	91	1			0.12
	21–25	121	0.28	0.07–1.11	0.07	
	**26–30**	**140**	**0.05**	**0.01–0.25**	**<0.01**	
	**31–35**	**106**	**0.11**	**0.02–0.53**	**<0.01**	
	36–40	81	0.35	0.06–2.11	0.25	
	**41–45**	**60**	**0.1**	**0.01–0.57**	**0.01**	
	missing	9	0.23	0.02–2.43	0.223	
**Sex**	Men	341	1			<0.001
	Women	267	1.42	0.49–4.12	0.52	
**In relationship**	Married/previous partner	377	1			0.02
	Always single	231	2.08	0.71–6.06	0.18	
Personal income	<£5000	82	1			0.15
	**£5000–£14999**	**149**	**0.19**	**0.05–0.69**	**0.01**	
	£15000–£24999	86	0.33	0.06–1.96	0.22	
	£25000–£34999	40	1.41	0.20–9.70	0.73	
	£35000–£49999	24	4.46	0.54–37.08	0.17	
	£50000–£74999	26	0.34	0.03–3.57	0.37	
	**>£75000**	**5**	**12.94**	**1.05–159.34**	**0.05**	
	**missing**	**196**	**4.17**	**1.24–13.96**	**0.02**	
Employment	Employed	310	1			0.07
	**Education**	**84**	**6.22**	**1.59–24.41**	**<0.01**	
	Unemployed	**79**	0.68	0.16–2.97	0.61	
	HW or sickness	135	2.08	0.62–6.97	0.23	
Place of birth	UK	268	1			0.06
	Pakistan	177	0.57	0.15–2.17	0.41	
	**Bangladesh**	**163**	**0.19**	**0.07–0.53**	**<0.01**	
Friends of same	**A lot/almost all**	**374**	**1**			0.02
ethnic group	**about half**	**137**	**0.41**	**0.13–1.30**	**0.13**	
	**a few/none**	**95**	**0.46**	**0.13–1.70**	**0.25**	
Language used at home	**English**	**195**	**1**			0.08
	**Other**	**413**	**0.32**	**0.11–0.97**	**0.05**	
Attendance to pray	Never	133	1			0.008
	< or = monthly	116	1.99	0.38–10.39	0.42	
	> or = weekly	355	1.08	0.38–3.02	0.89	
Discrimination	per point	608	0.79	0.31–2.05	0.63	0.002
Political engagement	per point	608	0.6	0.31–1.06	0.08	0.07
Life Events	per point	608	0.72	0.38–1.38	0.32	0.009
Social Capital (SC)	per point	608	0.86	0.62–1.20	0.36	0.01
SC-Satisfaction	per point	608	1.12	0.54–2.34	0.76	0.001
SC-Trust	per point	608	0.82	0.45–1.47	0.49	0.005
SC-Safety	per point	608	0.5	0.24–1.02	0.06	0.04
Depressive symptoms	per point	527	0.97	0.86–1.04	0.66	0.002
Anxiety symptoms	per point	562	0.97	0.87–1.09	0.64	0.002
Poor Health Score	0	411	1			
	1	60	0.51	0.13–2.0	0.33	0.07
	2	44	3.49	0.8–15.24	0.1	
	**3**	**57**	**0.08**	**0.02–0.35**	**<0.01**	
	**4**	**36**	**0.01**	**0.001–0.10**	**<0.01**	
Injuries A&E attendance	No	515	1			0.03
	Yes	93	3.29	0.90–12.07	0.07	

Those at greater risk of sympathies for *radicalisation* had an income of more than £75,000 but also included those who declined to give financial information, those in education rather than employment. Strikingly, many putative factors found in the literature did no show any associations, for example, social contacts, social capital, political influence, discrimination, frequency of visiting a mosque for prayers, gender, proportion of friends of the same ethnic background, and being single. One social capital measuring feels of safety fell short of statistical significance in univariate models.

The univariate analyses show a lower risk of having sympathies for *radicalisation* in the 26–35 and 41–45 age groups compared with the 18–20 age group; where total income is between £5000 and £14,999 compared with income of less than £5000; amongst people born in Bangladesh compared with those born in UK or Pakistan; amongst those speaking a language other than English at home; and amongst those with poorer general health.

The multivariate logistic regression model shows independent effects of each variable (overall R^2^ = 0.374, X^2^ = 77.11, N = 608, p<0.001). Using the same reference groups as in the univariate analyses, the following findings were significant. The findings of a higher risk of radicalisation amongst those on incomes of greater than £75,000 were sustained, however, the rather wide confidence interval suggests this is an imprecise estimate (OR = 44.14, 1.04–1871.67, p = 0.05). Those who scored 2 on poor health (compared with scoring 1) were at a slightly higher risk of radicalisation (OR = 4.12, 1.12–15.43, p = 0.03) whereas those scoring 3 or 4 were at lower risk (0.1, 0.02–0.53 and 0.01, 0.004–0.06), respectively suggesting an inverted U shape relationship with poor self-reported health. The language spoken at home was not entered as this showed co-linearity with place of birth. For a sensitivity test using just the six items in factor 1 as an outcome, we repeated the multivariate model. Although there was less power and the findings were therefore statistically not significant, the trends were identical and similar characteristics emerged as relevant risk and protective characteristics.

All those aged over 21 were at a lower risk of having sympathies for radicalisation (21–25 age group OR = 0.21, 0.04–0.94, p = 0.04; 26–30 age group: OR = 0.02, 0.002–0.1, p<0.01; 31–35 age group: OR = 0.05, 0.01–0.34, p<0.01). People with incomes of £5000 to £14,999 were also at lower risk (OR = 0.16, 0.03–0.91, p = 0.04) than those with lower incomes, as were those born in Bangladesh (OR = 0.19, 0.05–0.68, p = 0.01) compared with the UK.

## Discussion

### Findings in Context

This is the first study of common mental disorders and violent radicalisation, taking account of social and political attitudes, beliefs and health related behaviours associated with sympathies for radicalisation in a minority Muslim-heritage population sample of South Asian ethnic origin, living in Britain. It becomes clear that sympathies for terrorist acts are very rare, yet some individuals expressed strong support for serious acts. The prevalence of sympathies is equivalent to that found in the study of Muslim Americans [17] but much lower than found in Muslim majority countries [15], [16].

Two hypotheses have previously been proposed to explain sympathies for radicalisation. [14] First that social and health inequalities, poverty and discrimination, coupled with poor social networks, poor social capital and unemployment produce grievances. Secondly, that sympathies for radicalisation, are part of the radicalisation process, and their emergence is entirely a political process shaped by those in more influential positions and not related to health problems, poor socio-economic status, or mental health problems. [30] No previous UK based study has attempted to investigate the relationship between depressive and anxiety symptoms and sympathies for radicalisation and terrorism in a British Muslim sample; measuring anxiety and depressive symptoms is also important as these are known to be associated with suicidal ideas, poor health and premature mortality. [46], [47] The study showed that poor health or adverse experiences were not influential in radicalisation. This study does not support the view that sympathies for terrorist acts develop as a result of grievance related poor health (physical and mental) and social inequalities, or poor education or a lack of political engagement.

The trends suggest that people in education and high earners were more likely to support radical acts; this may reflect accident proneness and risk taking behaviour more generally, or this sub-group may have much in common with gang members who commit violent acts. [48] We did not have a measure of exposure to war and conflict, which may be important in radicalisation, however, we did assess life events including death, and assault and we also assessed recent injuries resulting in hospital visits. The association with recent injuries fell short of significance, and post hoc analysis of individual events showed that only those who had contact with the criminal justice agencies were more likely to show sympathies (OR = 23.45, 1.49–368.29), whilst loss events (job, friend) appeared not to show associations. Future studies should include detailed inventories of personality and risk-taking. However, screening of those who attend emergency departments is not justified. Those not reported poor health and those born in the UK showed greater levels of sympathy for terrorist acts and might be considered vulnerable to radicalising forces. Why poor health is associated with less support for radical causes remains unclear; perhaps illnesses and the limitations of functioning become the focus everyday lives, although the relationship is a complex one. Future work might establish the direction of these relationships and measure health status more objectively, and include detailed personality inventories to assess the role of crime, gang membership and personality disorders.

### Strengths and Weaknesses

The study shows it is possible to ask people about sympathies for radicalisation and terrorism in a manner that does not offend or lead to withdrawal. Such a measure permits testing of interventions in populations an assessment of how to engage those who might be disaffected. And there were no adverse incidents reported to our data independent monitoring committee.

Although we cannot infer that sympathies for violent protest and terrorism are necessarily linked to actual terrorist actions, it is clear that sympathies and uncertainty, as opposed to condemnation, are necessary ingredients for developing radicalised political viewpoints and terrorist actions may follow. The findings are proposed to be preliminary pending further study with larger samples. Although, we used questions to assess social capital rather than a validated scale, other indicators of exclusion using validated scales appeared to show negative findings; for example, those designed to assess discrimination and political engagement. Preventive interventions for violent radicalisation as defined by our measure direct attention to risk factors that do not easily align with the reduction of social and health inequalities. There may be sub-groups not represented in sufficient numbers within the population who are at higher risk of radicalisation, for whom social and health inequalities, and discrimination and political engagement are more important. A larger study including other ethnic, and cultural groups is required, along with further evaluation of our new measure of radicalisation of sympathies for violent protect and radicalisation.

### What is Known?

- Violent radicalisation is a social and psychological process by which people are influenced to take part in violent protest and terrorism
- There are several theories about what makes people develop sympathies for violent radicalisation and terrorism, the main ones being that these emerge due to grievances about social and health inequalities, discrimination, poverty, poor education, poor mental health, poor political engagement and attitudes to foreign policy are responsible
- There is little population research, and none that includes Muslim population samples in England, and their health status and their attitudes to radicalisation and terrorism.

### What this Study Adds

- Sympathies for violent radicalisation were uncommon
- Young healthy people, in education rather than employment, and born in the UK are more likely to sympathise with violent radicalisation and terrorist causes
- People with poor health, migrants, and older people were more likely to condemn violent radicalisation. Discrimination, poverty, social and health inequalities, political engagement and attitudes to foreign policy were not relevant.
